# A Web-Based Decision Aid for Caregivers of Persons With Dementia With Firearm Access (Safe at Home Study): Protocol for a Randomized Controlled Trial

**DOI:** 10.2196/43702

**Published:** 2023-01-31

**Authors:** Virginia McCarthy, Jennifer Portz, Stacy M Fischer, Emily Greenway, Rachel L Johnson, Christopher E Knoepke, Daniel D Matlock, Faris Omeragic, Ryan A Peterson, Megan L Ranney, Marian E Betz

**Affiliations:** 1 Department of Emergency Medicine School of Medicine University of Colorado Anschutz Medical Campus Aurora, CO United States; 2 Injury and Violence Prevention Center Colorado School of Public Health University of Colorado Anschutz Medical Campus Aurora, CO United States; 3 Division of General Internal Medicine School of Medicine University of Colorado Anschutz Medical Campus Aurora, CO United States; 4 Department of Biostatistics and Informatics Colorado School of Public Health University of Colorado Anschutz Medical Campus Aurora, CO United States; 5 Adult and Child Center for Outcomes Research and Delivery Science School of Medicine University of Colorado Anschutz Medical Campus Aurora, CO United States; 6 Division of Cardiology School of Medicine University of Colorado Anschutz Medical Campus Aurora, CO United States; 7 VA Eastern Colorado Geriatric Research Education and Clinical Center Rocky Mountain Regional VA Medical Center Aurora, CO United States; 8 Division of Geriatric Medicine School of Medicine University of Colorado Anschutz Medical Campus Aurora, CO United States; 9 Brown-Lifespan Center for Digital Health Brown University Providence, RI United States; 10 School of Public Health Brown University Providence, RI United States; 11 Department of Emergency Medicine Warren Alpert Medical School Brown University Providence, RI United States

**Keywords:** dementia, caregiver, firearms, decision aid decision-making, Alzheimers, ADRD, decision, storage, web-based

## Abstract

**Background:**

Firearm safety among individuals with Alzheimer disease and related dementias (ADRD) is an underdiscussed and underresearched concern in the United States, especially given the growing population of community-dwelling adults with ADRD. The “Safety in Dementia” (SiD) web-based decision aid was developed to support caregivers in addressing firearm access; the efficacy of SiD is unknown.

**Objective:**

Through the SiD decision aid, the Safe at Home (S@H) study aims to support caregivers in making decisions about home safety that align with their goals and values, and behaviors regarding firearm access for persons with ADRD and firearm access.

**Methods:**

The S@H study is a 2-armed randomized controlled trial to test the effect of the SiD decision aid on caregivers of community-dwelling adults with ADRD who have firearm access. S@H aims to recruit 500 ADRD caregivers (age ≥18 years, fluent in English or Spanish, and in the United States) through online or social media advertisements and through relevant organizations. Participants are randomized to view SiD or a control website at their own pace; all participants complete web-based questionnaires at baseline, 2 weeks, 2 months, and 6 months. The primary outcome is immediate preparation for decision-making; secondary outcomes include longitudinal decision outcomes and self-reported modifications to firearm access. The relative reach and effectiveness of each recruitment method (online/social media and through relevant organizations) will be assessed by examining differences in caregiver participation, retention rates, and relative cost.

**Results:**

The study enrollment began in May 2022. As of December 2022, a total of 117 participants had enrolled.

**Conclusions:**

The S@H study is the first randomized trial of a firearm safety decision aid for ADRD caregivers. The results from this study will inform how best to support caregivers in decision-making regarding firearm safety. Further, results may guide approaches for recruiting caregivers and for dissemination of resources.

**Trial Registration:**

ClinicalTrials.gov NCT05173922; https://clinicaltrials.gov/ct2/show/NCT05173922

**International Registered Report Identifier (IRRID):**

DERR1-10.2196/43702

## Introduction

### Background

Approximately 5.8 million Americans—roughly 10% of all adults aged 65 years or older, and 17% of Hispanic older adults [1]—live with Alzheimer disease and related dementias (ADRD) [2]. Almost three-quarters of people with ADRD live in the community [2] supported by an estimated 16 million informal caregivers (eg, spouses or other family members) [2]. Caregivers face myriad concerns and demands, including daily safety considerations, financial management, and long-term goals of care. There is an urgent need for effective, acceptable interventions to support informal caregivers in decision-making and behavior change to act on those decisions, especially regarding home safety. Ideally, the development and dissemination of such interventions—to the broad, diverse community of informal caregivers—could help reduce caregiving burden, delay nursing home placement, and support aging in place [3].

Many ADRD caregivers face difficult decisions about when and how to address the sensitive topic of in-home firearms. In the United States, an estimated 33%-60% of people with ADRD have a firearm in the home [4,5], and 38% of ADRD caregivers identify firearms as an issue to address [4]. Approximately one-third of older Americans (≥65 years) own a gun [6]; yet in a large sample of older firearm owners, only one-fifth reported having a plan for securing, removing, or transferring firearms if they could not handle them safely [7]. For firearm access, caregivers worry about how to take action, questions relate to legal and logistic uncertainties, and lack of guidance [8]. In a national survey of caregivers, although the majority supported ADRD-relevant firearm safety counseling by health care providers, only 5% had ever received it [9]. Caregivers must balance safety concerns (of both the person with ADRD and those around them) with considerations about the person with ADRD’s response (eg, anger) and logistics (eg, cost of various options).

Providing resources to caregivers regarding firearm safety is of particular importance due to increased risk of suicide and risk related to behavior changes associated with ADRD. In the United States, 70% of older adult suicides involve a firearm, and suicide risk is elevated among individuals with ADRD, especially in early ADRD [10-12]. The rate of suicide among individuals in a prospective cohort study following individuals in the 12 months after dementia diagnosis was 26.42 per 100,000 person years, totaling 705 deaths by suicide of which 63.3% (nearly 450 deaths) were completed by firearm [13]. Dementia also raises concerns about the safety of others given the behavioral changes that commonly co-occur with ADRD, including confusion and emotional volatility. Violence toward others occurs annually in approximately 1% to 3% of older adults with ADRD [14]. Regarding firearm safety, even among older adults with prior training and safe handling skills, unintentional (“accidental”) firearm injuries can also occur due to impairments in judgment, dexterity, and memory [9].

Web-based resources have the potential to reach a broad range of caregivers. More than 75% of American adults aged 30-64 years use at least one social media platform, most commonly Facebook and Instagram among middle-aged and older adults [15]. Web-based support groups are a leading source of health information for people living with or caring for loved ones with chronic disease [16,17]. The National Institute on Aging’s (NIA) Informal Caregiving Panel and Alzheimer’s Disease Research Summit recommended the development of technologies to integrate evidence-based treatments and support caregivers in the context of ADRDs [18-20]. Internet tools offer the promise of reaching diverse groups across broad geographic regions and allow individualized timing and pace of use [21]. eHealth interventions for informal ADRD caregivers show promise [22-24], are cost-efficient [25], and offer a way to sustain caregiver engagement over time [23]. The privacy afforded by eHealth, already cited as important by ADRD caregivers [23], may be particularly critical for the sensitive topic of firearms. ADRD caregivers may also prefer at-home internet resources or interventions because of limited ability or time to travel to an intervention site (eg, due to being with a home-bound person) [26].

### Prior Work

**Figure 1:**
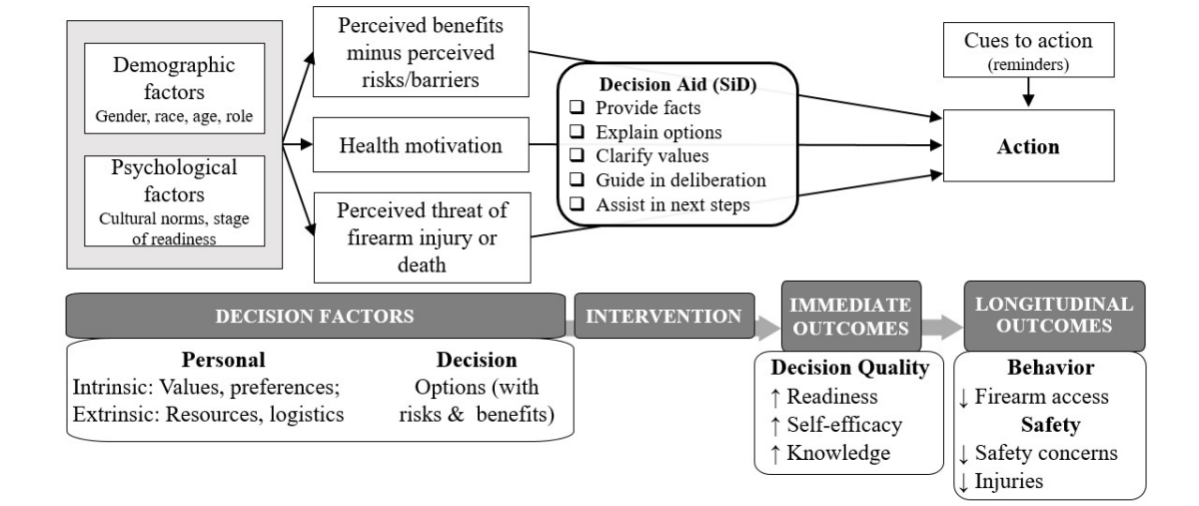
Theoretical framework of Safety in Dementia (SiD) immediate and long-term impact. Rectangles represent Health Belief Model. Ovals represent Ottawa Decision Support Framework and related measures.

To address caregiver needs related to firearm access, and guided by theory [27-29], our team previously developed the web-based Safety in Dementia (SiD) decision aid [30], which is available for public use at no cost [31]. This web-based tool adheres to the International Patient Decision Aid Standards [29] and recommendations for user-friendly web design for older adults [32-34]. SiD is based on behavior change and decision-making theories [27,28] (Figure 1) and includes content to help caregivers understand options (with a balanced presentation of benefits and drawbacks) and then be motivated to take the action that works best for their situation. SiD provides parallel sections regarding driving safety and general home hazards to broaden reach and contextualize firearm safety, and the tool was designed in collaboration with partners from the firearms community. The SiD tool was translated into Spanish using a rigorous translation process including independent back-translation to English by certified translators with adjudication of any discrepancies [35,36]. The pilot trial of SiD, in a national convenience sample of caregivers of community-dwelling people with ADRD, found 44% of caregivers reported safety concerns regarding firearm access and approximately 30% were currently considering options for what to do about firearm access [37].

### Goal of this Study

The primary goal of the S@H study is to assess the efficacy of the SiD decision aid on caregiver decision-making and behaviors regarding firearm access in the home of the person with ADRD. The study hypothesis is that the decision aid will increase the preparation for decision-making and the self-efficacy of informal caregivers to make and implement decisions that effectively address firearm access, thereby reducing firearm injury risk.

## Methods

### Study Design

The S@H study is a 2-armed randomized controlled trial. The study protocol follows the SPIRIT (Standard Protocol Items: Recommendations for Interventional Trials) Guidelines [38]. The trial will be conducted and reported according to 2 checklists: the CONSORT-EHEALTH (Consolidated Standards of Reporting Trials of Electronic and Mobile Health Applications and Online Telehealth; Multimedia Appendix 1) [39] and the SUNDAE (Standards for Universal Reporting of Patient Decision Aid Evaluation; Multimedia Appendix 2) [40].

### Theoretical Framework

The SiD decision aid and the S@H study are based on the Ottawa Decision Support Framework (Figure 1) [27], which incorporates concepts from psychology, social support, self-efficacy, and decision conflict. The framework suggests that decisional needs (eg, conflict/uncertainty, knowledge, and values) affect decision quality, with a decision being higher quality when it is informed by and reflective of the person’s values. Decision aids can increase decision quality by addressing decisional needs, by identifying the decision, by providing a balanced explanation of the risks and benefits of options, by helping clarify personal values, and by activating the individual for decision-making [29,41]. Decision aids improve communication and knowledge and decrease decisional conflict and regret [42]. Application of the Health Belief Model [28] further expands this theoretical framework for how decision aids encourage action.

### Eligibility

**Figure 2:**
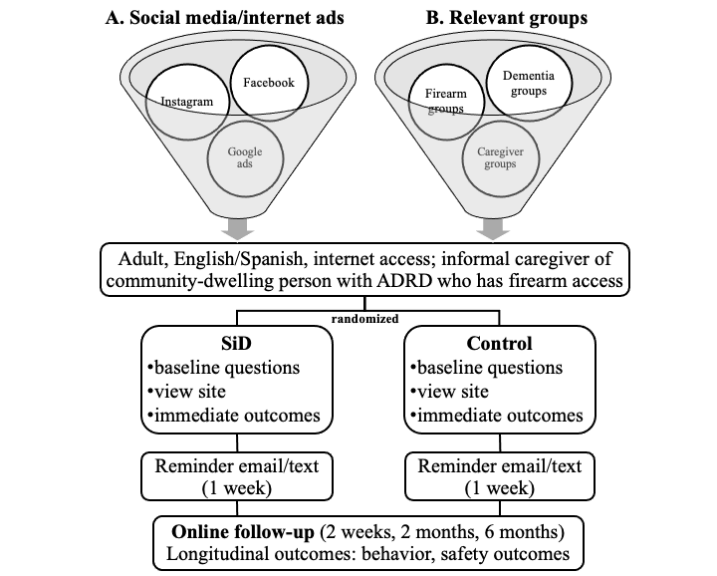
Study flow. ADRD: Alzheimer disease and related dementias; SiD: safety in dementia decision aid.

Participants must be adults aged 18 years or older, who live in the United States, are fluent in English or Spanish, and identify as informal caregivers for a community-dwelling person with ADRD who has firearm access. “Community-dwelling” is defined as a person with ADRD living in a private home and not in any type of caregiving facility (Figure 2). Participants must have internet access but do not need to live in the same home (or state) as the person with ADRD. Those in legal custody or institutionalized are not eligible to participate.

### Recruitment

The S@H study aims to enroll 500 English- and Spanish-speaking caregivers through social media recruitment (including Facebook, Instagram, and Google ads) and relevant organizations in dementia/caregiver support, aging, and firearms. Social media postings are designed by the study team and then enhanced, implemented, and tracked by a digital marketing organization. Social media advertisement campaign parameters are set and modified to maximize reach and click-throughs, per standard web-based advertising practice; advertisements are iteratively adjusted for adequate gender and age diversity throughout the study.

Recruitment is also conducted via direct email through relevant organizations in dementia/caregiver support, aging, and firearm organizations, including web-based research databases like ResearchMatch and TrialMatch. Direct personal contact is made with relevant organizations by the study team and through the guidance and professional relationships of the Expert Advisory Panel. Study staff post advertisements directly (eg, in ResearchMatch) or send organizational representatives a toolkit with a sample text for inclusion in organization-generated newsletters, listservs, and flyers, on websites, and so on; sample social media content; and social shareables (images and informational graphics). The research team follows up 1 week after sending the media toolkit to inquire about the use of materials and to provide support as needed.

All advertisements contain similar messages and direct individuals to a landing webpage with a description of the study and identification of the lead organization (University of Colorado). The study has separate, identical landing web pages for each recruitment method and subgroup to allow tracking of overall analytics and participation by recruitment modality. There are 12 landing pages in total: 6 pages in English and 6 pages mirrored in Spanish. At each landing page, interested individuals can complete a brief web-based eligibility form. Those who choose to stop participation at any time or who are ineligible are provided with the hyperlink for the control (the NIA Home Safety Checklist for Alzheimer’s Disease website, described below [43]).

Potential participants who are eligible based on the web-based form provide their contact information, and study staff then contact them by phone to validate identity (to reduce fraud or bot enrollment) and review eligibility and informed consent (Multimedia Appendix 3). For eligible, interested individuals, a study team member performs the randomization in REDCap (described below) [44,45] and emails or texts, depending upon preference indicated by the participant, the web-based survey link for the baseline assessment. Participants complete the survey at a time and in a place of their choosing, on a computer or other web-enabled device, and at their own pace, allowing them to stop and come back to the survey.

Participants complete a set of web-based questionnaires before viewing the SiD decision aid or the control website (Figure 2, Table 1). After reviewing the website for as long as they want, participants complete a second set of web-based questionnaires with questions about the preparation for decision-making, self-efficacy, and knowledge (Table 1). One week after the baseline session, participants receive a text or email reminder (depending on their preference) with a hyperlink to the website to which they were randomized so they may visit it again if desired. Subsequent follow-up (at 2 weeks, 2 months, and 6 months) occurs via web-based surveys that are sent to participants. Participants receive incentives for each completed study survey (US $40 for baseline, US $60 for 2 weeks, and US $40 each for 2 and 6 months).

The study flow follows the SPIRIT template of recommended content for the schedule of enrollment, interventions, and assessments [38].

**Table 1:** Study flow for caregivers.

				Timepoint^a^ (months)										
				Enrollment		Allocation		Postallocation						Close-out
				–*t*		*t*_0_		*t*_0_		*t*_2 weeks_		*t*_2 mo_		*t*_6 mo_
**Enrollment**														
	Web-based eligibility screen			✓										
	Phone screen by staff			✓										
	Informed consent			✓										
	Randomization					✓								
**Interventions**														
	Safety in dementia or control website					✓								
**Assessments**														
	**Immediate outcomes**													
		Preparation for decision-making					✓		✓		✓		✓	
		Decision self-efficacy scale			✓				✓		✓		✓	
	**Longitudinal outcomes**													
		Action to reduce firearm access			✓				✓		✓		✓	
		Firearm access			✓				✓		✓		✓	
	**Subgroups**													
		Firearm injury/near-injury			✓				✓		✓		✓	
		Zarit caregiver burden							✓					
		Positive aspects of caregiving scale							✓					
		Stage of decision-making			✓				✓		✓		✓	
	**Other covariates**													
		PWD^b^ demographics			✓									
		PWD characteristics			✓				✓		✓		✓	
		Revised memory and behavior checklist			✓				✓		✓		✓	
		Caregiver demographics					✓							
		AD8^c^ Dementia severity			✓									
		Social media usage							✓					

^a^–*t*: pre-screening; *t*_0_: baseline survey; *t*_2 week_: 2-week survey; *t*_2mo_: 2-month survey; *t*_6mo_: 6-month survey.

^b^PWD: person with dementia.

^c^AD8: aging and dementia 8-item scale.

### Randomization and Blinding

Enrolled participants are randomly assigned in equal numbers to the SiD intervention or control arm (Figure 2). Randomization is stratified between recruitment group A (social media or internet) and group B (relevant organizations). To reduce bias and aim for balance across arms, participants are randomized into blocks of undisclosed size. The team biostatistician conceals allocation using a centralized, computer-generated list in REDCap [46]. The study team’s principal investigator and one of the biostatisticians are blinded; other members of the study team are unblinded. Participants are blinded to their study arm assignment.

### Description of Intervention

In the intervention arm, participants are directed via hyperlink to the SiD decision aid. Participants may view the site in the format they prefer (eg phone, computer, and tablet) and are recommended to view the site for at least 5-10 minutes but may view it for as long as they want. Like other decision aids, SiD guides the participant through the decision process to promote person-centric consideration of values and preferences [47].

### Description of Control

In the control arm, participants are directed via hyperlink to the NIA Home Safety Checklist for Alzheimer’s Disease website [43]. Similar to the intervention arm, participants in the control arm may view the site in the format they prefer (eg, phone, computer, and tablet). The control (NIA website) represents typical care, as it is an easily accessible website that consistently appears in the top 5 results of a Google search for “home safety dementia” and provides basic education about home safety in the context of dementia [43]. The checklist provides limited guidance on firearms (“*Lock up or remove these potentially dangerous items from the home: ... Guns and other weapons, scissors, knives, power tools, and machinery*”) [43] without information about specific locking or disposal options. Unlike the intervention, the NIA website does not guide the individual through the decision or promote person-centric consideration of values and preferences, making it an appropriate control [47].

### Primary and Secondary Outcome Measures

#### Primary Outcome

The primary outcome is the effect of SiD on preparation for decision-making, a core element of the Ottawa Decision Support Framework [27] as a precursor to behavior change (Figure 2). A high-quality decision is defined as an informal caregiver making an informed decision consistent with their values [48,49]. The Preparation for Decision-Making Scale assesses the perception of how useful a decision aid is in preparing for subsequent decision-making. Scores range from 1 to 5, calculated from the average of 10 constructs (each ranging from 1: “strongly disagree” to 5: “strongly agree”). The scale has total test reliability of 0.944 [50].

A secondary immediate outcome is decision self-efficacy (self-confidence or belief in one’s capability to make decisions), measured using the decision self-efficacy scale [51], as decision aids typically increase self-efficacy [52]. Transformed scores range from 0 (extremely low) to 100 (extremely high self-efficacy). In the SiD pilot trial, participants had a mean score of 77.6 (SD 17.2) after viewing the SiD firearm section [37] (Table 1).

#### Longitudinal Outcomes

Additional outcomes, also linked to our theoretical framework (Figure 1), are in Table 1. Firearm access (and actions to reduce access) for the person with ADRD is assessed with existing multipoint scales [52,53], with binary categorization (any access to ≥1 firearm versus no access to any firearms) and progression toward reduced access (eg, locking of additional firearm). This allows the identification of smaller, albeit important, changes. Firearm injury or near-injury in the person with ADRD or others in their home is assessed through questions about recent experiences (including incidents of threats with a firearm or “near-misses”) and perceived risks. Caregiver burden is measured by the short-form (3-item) Zarit Burden Interview [24,54,55]. Benefits of caregiving are measured by the Positive Aspects of Caregiving Scale [56-58]; scores range from 0 to 36, calculated from the sum of 9 items (measured on a 5-point scale ranging from 0: “disagree a lot” to 4: “agree a lot”), with higher scores indicating positive experiences.

#### Other Covariates

Caregiver characteristics include basic demographic characteristics like participation language (English/Spanish) age, gender, race/ethnicity, living situation (marital status, income, and urban/rural), and education. The characteristics of a person with ADRD (as reported by the caregiver) include age, gender, race/ethnicity, and living situation (marital status, income, and urban/rural). Dementia severity and course over time will be measured using the validated caregiver-reported aging and dementia 8-item scale Dementia Screening and Revised Memory and Behavior Checklist. The characteristics of the caregiver relationship to person with ADRD include the type of relationship (eg, spouse and nonrelated friend) and living situation (together or apart; if apart, proximity).

Study measures pertaining to the stages and quality of decision-making are based on validated measures currently available in the literature. Validated measures in Spanish were available for more than half of the English measures. For measures which were not available in Spanish, translation followed a forward and back translation process used throughout the study, incorporating similar phrasing and structure of other validated measures when possible.

#### Additional Measures

The approximate cost per enrolled participant will be calculated based on advertising costs and fees, and staff time. Website analytics will also be analyzed for each recruitment group and subgroup randomized to SiD, measures will include average time spent on SiD (mean and SD, median, and IQR) and pages viewed.

#### Data Monitoring

The study team will work with a Data Safety Monitoring Board, along with the IRB and the NIA, to monitor participant safety, evaluate study progress, review procedures for maintaining the confidentiality of data, and ensure the quality of data collection, management, and analyses. The following adverse and serious adverse events are monitored: any kind of negative interaction from intervention (eg, verbal disagreement or physical fight), increased stress due to actions taken to reduce firearm access for a person with dementia, physical injury sustained during actions to reduce firearm access for a person with dementia, suicide ideation or attempt in caregiver or individual with dementia, hospitalization due to emotional factors (stress, depression, suicidal, or homicidal ideation or intent), or hospitalization or surgery for a physical injury sustained during actions to reduce firearm access for a person with dementia. Each adverse event is graded by severity and relationship to intervention.

#### Data Collection

All participant-level data are collected and stored using self-reported, web-based questionnaires through REDCap [22,44,45]. Additional data are obtained through analytic reports provided by the digital marketing firm for each of the unique landing pages and web-based eligibility forms, while these sites will be identical, they will separate the trial participants by their origin of enrollment (group and subgroup). Website analytics will be analyzed at the level of groups or subgroups, as it will not be possible to identify individual participants from their website usage (eg, they do not login to a site or have other markers of identity). For social media and internet advertisements, ad impressions, click-throughs, and enrollment will be tracked and analyzed.

### Statistical Analysis

Descriptive statistics will be computed for baseline caregiver characteristics (including key covariates, Table 1), reporting on differences between (1) intervention arm, (2) follow-up status, and (3) recruitment modality. To test the primary hypothesis that SiD increases immediate decision quality compared to control, we will test for a difference in the adjusted mean Preparation for Decision Making score at baseline after viewing SiD or control using multiple linear regression (MLR), with adjustment for baseline characteristics (eg, age, gender, caregiver burden, and severity of ADRD). Other components (decision quality, decision self-efficacy, and knowledge) will be analyzed similarly. To assess longitudinal end points, linear mixed models will replace multiple linear regression to assess changes in self-efficacy and knowledge over time by treatment arm, allowing an interaction of treatment arm and time [59,60]. Substantial efforts will be made to minimize missing data in data collection, and our models will adjust for covariates associated with drop-out to attenuate bias. If necessary, we will also use methods such as multiple imputation, pattern mixture models, or sensitivity analyses, following intent-to-treat principles [61].

To test the secondary hypothesis that SiD increases the odds that action is taken to reduce firearm access, a binomial-family logit-link generalized linear mixed models will be used with subject-specific random effects to account for correlations across measurements from the same individual and with fixed effects to adjust for baseline characteristics. The interaction between intervention and time will be tested to assess if the effect of SiD varies over time. We will use interaction terms to identify potential differential effects of treatment according to age, whether the caregiver lives with the person with ADRD (vs not), and recruitment group. Exploratory heterogeneity of treatment effect will also be conducted by caregiver gender, urban versus rural residence, and language (English vs Spanish). Additionally, we will examine longitudinal change in caregiver burden and reported safety concerns, and how these might affect treatment effects. Both multiplicity-adjusted and unadjusted *P* values will be presented for these comparisons.

We will use analytics from the eligibility screening websites to estimate the proportion of individuals who were eligible by recruitment modality, including separately by language, the proportion of eligible individuals who enrolled, and the rate of recruitment. The cost of recruitment per participant will be calculated based on advertising costs, fees paid to organizations, and estimates of study staff time [62,63]. Finally, among SiD participants, we will compare minutes on-site and trial retention at 6 months across recruitment modalities. Covariates will be included in multivariable models if they are associated with dropout, hypothesized a priori to be adjusted for, or if their inclusion improves the overall model’s Akaike information criterion. If there is evidence that normality assumptions have been violated, appropriate transformations will be used, or the appropriate link function (eg, logit link for dichotomized measures).

#### Power

The target sample size for this study (n=500), which includes recruitment via social media (n=250) and relevant organizations (n=250), was powered conservatively for the primary outcome to allow comparisons by recruitment groups and other participant characteristics. In the pilot trial of 15 caregivers, the mean Preparation for Decision Making scores (using 9 constructs) was 3.9 (SD 0.7) after SiD versus 3.6 (SD 0.2) in the control group; this corresponds to a standardized effect size of 0.42 (3.9 vs 3.6 with a pooled SD of 0.7; *d*=0.3/0.7=0.42) [37,64]. Based on a *t* test, with the proposed sample size of 500 participants, the analysis will be able to detect a *d*=0.42 effect size with a very high power of >99% (α=.05) between SiD and control for the primary outcome (preparation for decision-making). Even if the effect is smaller (eg, *d*=0.30) and the sample size is 80% (400 in total), the power remains high at 84.9%. With a sample size of 500, there will be 125 in each of the final four analytic groups (stratified by recruitment group A vs B, SiD vs control). This sample will yield 80% power to detect any subgroup-specific standardized effect of *d*=0.36 or greater between SiD and control, including by recruitment group and other participant characteristics (using a *t* test and assuming sample sizes of 125 in each subgroup). Power calculations were computed using R version 4.0.2 (R Foundation for Statistical Computing) [65].

### Ethics Approval

This study was approved by the Colorado Multiple Institutional Review Board (COMIRB) (21-4084). All study procedures and documents were reviewed and approved under Expedited Review, Category 7, by COMIRB. COMIRB approved a waiver of written documentation of informed consent and Health Insurance Portability and Accountability Act authorization. The S@H trial is registered with ClinicalTrials.gov (ClinicalTrials.gov NCT05173922).

## Results

This study was funded in December 2021. Data collection began in May 2022. As of December 2022, 117 participants have been enrolled in the study. This study will test the efficacy of the SiD web-based decision aid in decision-making among ADRD caregivers. Anticipated findings from this study include participant reports of increased preparation for decision-making and greater alignment of decision-making with their goals and values for all study participants; a higher degree of change is anticipated among participants randomized into the SiD decision aid arm. Further, this study anticipates effects on longitudinal decision outcomes including self-reported modifications that caregivers have made to firearm access for persons with ADRD. This study will also contribute to knowledge regarding effective tools for web-based recruitment methods, especially for ADRD caregivers.

## Discussion

### Principal Findings

This study aims to address the need for access to effective resources to support ADRD caregivers in decisions about firearm safety. Caregivers make important decisions regarding ADRD care, including home safety. Additional guidance—such as through SiD, if it is found effective—could help align decisions with caregiver goals and values, especially regarding difficult and under-discussed topics such as firearm injury prevention.

### Strengths and Limitations

Collaborative, nonjudgmental approaches to firearm injury prevention show great promise. A major strength of the SiD decision aid and this study is that they were developed with ideas and recommendations of individuals from the firearms community, including range owners, retailers, and firearm instructors. Such early—and ongoing—stakeholder engagement will help optimize intervention acceptability and effectiveness [66]. Many firearm owners have strong beliefs in self-determination and individual liberty [67,68], which are reflected in many of the same philosophical tenets underpinning shared decision-making more broadly [69]. A culturally sensitive intervention may allow caregivers to more fully and autonomously explore options [70]. An intervention that is responsive to cultural factors and personal values may also have higher acceptability and uptake than historical approaches to promoting firearm safety, which are clinician-delivered and include “one size fits all” recommendations to all firearm users. SiD is the first such tailored intervention for firearm safety in ADRD.

The varied methods of recruitment of ADRD caregivers across the United States is another strength of this study. The prevalence of digital-only or digital/in-person hybrid trials has increased during COVID-19. However, the effectiveness and representativeness of digital recruitment remain under debate. In particular, for sensitive issues like ADRD and firearm safety, it is essential to rigorously evaluate the success and equity of novel recruitment methods. The final sample may not generalize to all ADRD caregivers, given the language restriction (English and Spanish) and the need for access to and ability to use web-based technology. Those who participate in a study may also be different from the general population of ADRD caregivers.

### Future Directions

“Designing for dissemination” in interventions can promote real-world use [71]. The SiD decision aid and study design were guided by a desire to disseminate effective, acceptable interventions to support diverse caregivers, and reduce firearm injury among older adults. Translation into real-world application is central to the research design of the S@H study, which aims to evaluate tools and processes that are concurrently easy to implement and responsive to the needs of all involved [72], from caregivers who might use the tool, to older adults with ADRD themselves, to service providers who may recommend it to families, to firearm retailers and advocacy groups seeking to educate their communities [71,73]. Should the tool prove effective in the study, future work should examine its dissemination through formal and informal channels.

### Conclusions

The S@H study is the first national randomized trial to evaluate a firearm safety resource for ADRD caregivers. This study will provide information on the efficacy of the SiD decision aid as well as insights on how to recruit caregivers in a national, web-based trial.

## Appendix

Multimedia Appendix 1 CONSORT-eHEALTH (V 1.6.1) checklist.

Multimedia Appendix 2 SUNDAE (Standards for Universal Reporting of Patient Decision Aid Evaluation) Checklist for evaluation studies of patient decision aids.

Multimedia Appendix 3 Informed consent language.

Multimedia Appendix 4 External grant review from the Firearm Injury and Mortality Prevention Research - National Institute on Aging (National Institutes of Health, USA).

## Abbreviations

ADRD: Alzheimer disease and related dementias
COMIRB: Colorado Multiple Institutional Review Board
CONSORT-EHEALTH: Consolidated Standards of Reporting Trials of Electronic and Mobile Health Applications and Online Telehealth
NIA: National Institute on Aging
S@H: safe at home study
SiD: safety in dementia decision aid
SPIRIT: Standard Protocol Items: Recommendations for Interventional Trials
SUNDAE: Standards for Universal Reporting of Patient Decision Aid Evaluation

## Data Availability

Requests for data from other researchers and the public will be considered, and data will be made available in accordance with local institution policies, Institutional Review Board (IRB) recommendations, local/state/federal laws and regulations, and considerations for publication. Any applicable data sharing will follow Health Insurance Portability and Accountability Act rules.
