# Supplementary material for: A Web-Based Decision Aid for Caregivers of Persons With Dementia With Firearm Access (Safe at Home Study): Protocol for a Randomized Controlled Trial
Source: JMIR Res Protoc. 2023 Jan 31;12:e43702. doi: 10.2196/43702 (PMC9929727; doi:10.2196/43702)
Supplement: Multimedia Appendix 2 [file resprot_v12i1e43702_app2.pdf]

SUNDAE Checklist for evaluation studies of patient decision aids

Section/Topic	Page No.	Item No.	Checklist Item
**Title and Abstract**			
	abstract	1	Use the term patient decision aid in the abstract to identify the intervention evaluated and, if possible, in the title.
	abstract	2	In the abstract, identify the main outcomes used to evaluate the patient decision aid.
**Introduction**	**As part of standard introduction (the problem, gaps, purpose)**		
	2-3	3	Describe the decision that is the focus of the patient decision aid.
	3	4	Describe the intended user(s) of the patient decision aid.
	3	5	Summarize the need for the patient decision aid under evaluation.
	3	6	Describe the purpose of the evaluation study with respect to the patient decision aid.
**Methods**	**Studies with a comparator should also address Items 7-13 for the comparator if possible**		
	3-4	7	Briefly describe the development process for the patient decision aid (and any comparator), or cite other documents that describe the development process. At a minimum include:<br>• Participation of stakeholders in its development<br>• The process for gathering, selecting and appraising evidence to inform its content<br>• Any testing that was done
	7	8	Identify the patient decision aid evaluated in the study (and any comparator) by including:<br>• Name or information that enables it to be identified<br>• Date and/or version number<br>• How it can be accessed, if available
	7	9	Describe the format(s) of the patient decision aid (and any comparator) (e.g. paper, online, video).
	3	10	List the options presented in the patient decision aid (and any comparator).
	3	11	Indicate the components in the patient decision aid (and any comparator) including:<br>• Explicit description of the decision*<br>• Description of health problem*<br>• Information on options and their benefits, harms, and consequences*<br>• Values clarification (implicit or explicit)*<br>• Numerical probabilities<br>• Tailoring of information or probabilities<br>• Guidance in deliberation<br>• Guidance in communication<br>• Personal stories<br>• Reading level or other strategies to help understanding<br>• Other components<br>*These components are needed to meet the definition of a patient decision aid.
	3	12	Briefly describe the components from Item 11 that are included in the patient decision aid (and any comparator) or cite other documents that describe the components.

Section/Topic	Page No.	Item No.	Checklist Item
**Methods (cont.)**	7	13	Describe the delivery of the patient decision aid (and any comparator) including:<br>• How it was delivered (e.g. by whom and/or by what method)<br>• To whom it was delivered<br>• Where it was used<br>• When it was used in the pathway of care<br>• Any training to support delivery<br>• Setting characteristics and system factors influencing its delivery
	7-9	14	Describe any methods used to assess the degree to which the patient decision aid was delivered and used as intended (also known as fidelity).
	7-9	15	Describe any methods used to understand how and why the patient decision aid works (also known as process evaluation) or cite other documents that describe the methods.
	4	16	Identify theories, models or frameworks used to guide the design of the evaluation and selection of study measures.
	6-9	17	For all study measures used to assess the impact of the patient decision aid on patients, health professionals, organization, and health system:<br>• Identify the measures<br>• Indicate the timing of administration in relation to exposure to the patient decision aid and health care interventions
	6-9	18	For any instruments used:<br>• Name the instrument and the version (if applicable)<br>• Briefly describe the psychometric properties, or cite other documents
**Results**	**In addition to standard reporting of results**		
	NA	19	Describe the characteristics of the patient, family, and carer population(s) (e.g. health literacy, numeracy, prior experience with treatment options) that may affect patient decision aid outcomes.
	NA	20	Describe any characteristics of the participating health professionals (e.g. relevant training, usual care vs. study professional, role in decision making) that may affect decision aid outcomes.
	NA	21	Report any results on the use of the patient decision aid:<br>• How much and which components were used<br>• Degree to which it was delivered and used as intended (also known as fidelity)
	NA	22	Report relevant results of any analyses conducted to understand how and why the patient decision aid works (also known as process evaluation).
	NA	23	Report any unanticipated positive or negative consequences of the patient decision aid.
**Discussion**	**As part of the standard discussion section (summary of key findings, interpretation, limitations and conclusions):**		
	NA	24	Discuss whether the patient decision aid worked as intended and interpret the results taking into account the specific context of the study including any process evaluation.
	10-11	25	Discuss any implications of the results for patient decision aid development, research, implementation, and theory, frameworks or models.
**Conflict of Interest**			
	12	26	All study authors should disclose if they have an interest (professional, financial or intellectual) in any of the options included in the patient decision aid or a financial interest in the decision aid itself.
