# Supplementary material for: A Web-Based Decision Aid for Caregivers of Persons With Dementia With Firearm Access (Safe at Home Study): Protocol for a Randomized Controlled Trial
Source: JMIR Res Protoc. 2023 Jan 31;12:e43702. doi: 10.2196/43702 (PMC9929727; doi:10.2196/43702)
Supplement: Multimedia Appendix 3 [file resprot_v12i1e43702_app3.docx]

SiD R01 – Aim 1 Recruitment Call Log & Phone Screening & Consent Language (ENGLISH)

Today’s date: _______

Time of day:

□ Morning

□ Afternoon

□ Evening

□ Weekend

Who is making the call? [PRA’s name]

Hello, my name is _______ and I'm calling from the Safe at Home study at the University of Colorado. May I please speak with [participant’s name]?

***[If participant is not available/doesn’t answer (ie. VM script)]*** Hello, this is [research staff’s name] calling from the Safe at Home study at the University of Colorado. Thank you for completing the online form to join the study. We are grateful you are interested in participating. I’m reaching out to you to schedule a brief phone call to see if you are eligible for our study. Please give me a call back at [research staff’s office number]. Thank you!

***[If participant is available]*** Thank you for completing the online form to join the study. We are grateful you are interested in participating. I'd like to ask you a few more questions to see if you are eligible for our study.

***[If the participant indicates (at any time) that they are not interested]*** Thank you for considering this opportunity. May I ask why you are not interested in participating?

1. Are you 18 years of age or older?

□ Yes (1)

□ No (0) - The participant is INELIGIBLE, but still show the next question

1. Do you primarily reside in the United States?

□ Yes (1)

□ No (0) - The participant is INELIGIBLE, but still show the next question

***[If YES to “Other than this study, will you be participating in another University of Colorado research study within the same calendar year?”]***

We want to thank you for your time that you spend filling out the surveys. We would like to send you compensation for each survey you complete. We have to collect a tax form for compensation. Even if you are not a US citizen, we need to complete these tax forms. You do not need to be a US citizen to be in the study or to receive compensation for participation in the study.

1. Do you have a Social Security Number (SSN)?

□ Yes (1)

□ No (0)

***[If YES]***

We have to collect your SSN to complete the tax forms. This form is a W9 and you will fill in your SSN. This W9 form will be secured electronically and will be kept strictly confidential.

In order to receive compensation for participation, we have to have a W9 form for the University's tax reporting obligations. Your SSN will only be used for the W9 form and nothing else.

***[If NO]***

You can still participate if you don't have a SSN.

We will ask you to fill out the W-8BEN form. Your compensation for participation in the study will have 30% deducted for government taxes. This W-8BEN form will be secured electronically and will be kept strictly confidential.

In order to receive compensation for the study, we have to have a W-8BEN form for the University's tax reporting obligations.

1. Are you currently a caregiver of someone with dementia or Alzheimer's?

By caregiver, we mean someone (typically a family member or friend, sometimes with a power of attorney) who helps make decisions for the person with dementia about medical, financial, housing or other needs. We do not mean someone who is paid (as someone employed by a family or facility) to provide this care.

□ Yes (1)

□ No (0) - STOP

1. Does the person with dementia primarily reside in the United States?

□ Yes (1)

□ No (0) - STOP

1. Does the person with dementia live in a care facility? (for example, a nursing home or an assisted living facility)

□ Yes (1) - STOP

□ No (0)

1. Is the person with dementia able to access or use any firearms? This includes by themselves or when with another person, for any reason.

□ Yes (1)

□ No (0) - STOP

***[If not eligible]*** Thank you for your time and interest. Based on your responses, you are not eligible to participate in this research study. I appreciate you taking the time and talking with me today.

If you are interested in resources for caregivers of people with dementia, please visit the National Institute on Aging's Home Safety Checklist for Alzheimer's Disease.

***[If eligible]*** Based on your responses, you are eligible to participate in this research study. Do you want to move forward?

□ Yes (1)

□ No (0)

[If No] Ask the question: "Thank you for considering...”

[If Yes] Great! Now I will explain the study and read to you our consent form. Please stop me at any time if you have any questions. Based on your responses, you are eligible for participation in this research study (COMIRB #21-4048). Your decision to complete this study is completely voluntary. If you decide to participate, you may stop participating at any point.

By completing the study questionnaires, you are consenting to participate and agreeing for your responses to be used for research purposes.

If you agree to participate in this research study, you will be asked to:

- Complete an initial online questionnaire that’s divided into two sections: one right before viewing an online resource, and one right after viewing the online resource
- View an online resource about safety for people with dementia
- Complete a second questionnaire approximately two weeks after you complete the initial questionnaire
- Complete a third questionnaire approximately two months after you complete the initial questionnaire
- Complete a fourth questionnaire approximately six months after you complete the initial questionnaire

Between the first and second section of the initial questionnaire, you will be randomly assigned to view one of two websites. This study will have 2 different groups of research subjects like you. To decide which group you will be in, we will use a method of chance. This method is like flipping a coin or rolling dice. Each group will get slightly different information.

Both groups will view websites that include resources for caregivers of people with dementia. You will receive an email or text reminder one week after your initial questionnaire with a link to the online resource you viewed.

We expect that each questionnaire will take about 15-20 minutes to complete. You are free to skip any question you wish. You can decide to stop participating at any point in time.

If you complete the first two sections of the initial online questionnaire, you will be provided with $40. If you complete the second questionnaire approximately two weeks after you complete the initial questionnaire, you will be provided with $60. If you complete the third questionnaire approximately two months after you complete the initial questionnaire, you will be provided with $40 . If you complete the fourth questionnaire approximately six months after you complete the initial questionnaire, you will be provided with $40. Before providing you with compensation, we are required to collect a form from you. Please consult with your tax advisor to determine whether this compensation is taxable.

Your answers will be kept confidential. While the researchers will take every effort to ensure your confidentiality (through securing responses with a passcode and assigning you a unique number as an identifier), there exists a small possibility that someone outside of the researchers could view your personal information.

We will keep your participation in this research study confidential to the extent permitted by law. However, it is possible that other people may become aware of your participation in this study. For example, the following people/groups may inspect and copy records pertaining to this research.

- The Office of Human Research Protections in the U.S. Department of Health and Human Services
- The University of Colorado Institutional Review Board (a committee that reviews and approves research studies) and
- The University of Colorado Human Subjects Protection Office
- The National Institutes of Aging, the study sponsor

Some of these records could contain information that personally identifies you. Reasonable efforts will be made to keep the personal information in your research record private and confidential but absolute confidentiality cannot be guaranteed.

Feel free to contact the researchers with any questions by emailing them at [PRA email address]. You may have questions about your rights as someone in this study. If you have questions, you can call the COMIRB (the responsible Institutional Review Board). Their number is (303) 724-1055.

The data we collect will be used for this study but may also be important for future research. Your data may be used for future research or distributed to other researchers for future study without additional consent if information that identifies you is removed from the data.

If you would prefer not to participate in this research study but wish to view resources for caregivers of people with dementia, please visit the [National Institute on Aging’s Home Safety Checklist for Alzheimer’s Disease](https://www.nia.nih.gov/health/home-safety-checklist-alzheimers-disease).

Now, please answer the following Yes/No questions designed to help ensure you understand your rights as a research participant. If you provide an incorrect response I will read the correct answer.

Q1 Participating in this research study is entirely voluntary. I can choose to participate (or not), and withdraw from participating at any point. My responses will be used for research purposes.

□ Yes (1)

□ No (0)

*If response is No, the following text is displayed:* Correct answer is: Participating in this research study is entirely voluntary. You can choose to participate (or not), and withdraw from participating at any point. Your responses will be used for research purposes.

Q2 I will be asked to review an online resource and complete four online questionnaires if I choose to participate in this research study. Completing the questionnaires will require internet access via a computer, laptop, or tablet.

□ Yes (1)

□ No (0)

*If response is No, the following text is displayed:* Correct answer is: If you choose to participate, you will be asked to review an online resource and complete four online questionnaires.

Q3 I will be provided with $40 after completing the first questionnaire, $60 after completing the second questionnaire, $40 after completing the third questionnaire, and $40 after completing the fourth questionnaire,

□ Yes (1)

□ No (0)

*If response is No, the following text is displayed:* Correct answer is: If you choose to participate you will be provided with $40 after completing the first questionnaire, $60 after completing the second questionnaire, $40 after completing the third questionnaire, and $40 after completing the fourth questionnaire.

Q4 Any information I provide will be kept confidential and securely stored by the researchers.

□ Yes (1)

□ No (0)

*If response is No, the following text is displayed:* Correct answer is: The researchers will take every effort to ensure your confidentiality and store your research responses securely.

Great! The next step is that I will email you the link to complete the first survey. I will send you this email shortly.

Just to confirm, is this a good email address for you?: *[participant’s email address that was provided in initial screening form]*

***[If NO for “Other than this study, will you be participating in another University of Colorado research study within the same calendar year?]*** After completing your survey, you can expect to receive your Amazon gift card within a week.

***[If YES for “Other than this study, will you be participating in another University of Colorado research study within the same calendar year?” AND participant has SSN]*** After completing your survey, you can expect to receive your Amazon gift card within a week.

***[If YES for “Other than this study, will you be participating in another University of Colorado research study within the same calendar year?” AND participant doesn’t have SSN]*** After completing your survey, you can expect to receive the compensation within a month.

Thank you for taking the time and talking with me today. If you have any questions please don’t hesitate to reach out by phone or email.
