# Supplementary material for: A Web-Based Decision Aid for Caregivers of Persons With Dementia With Firearm Access (Safe at Home Study): Protocol for a Randomized Controlled Trial
Source: JMIR Res Protoc. 2023 Jan 31;12:e43702. doi: 10.2196/43702 (PMC9929727; doi:10.2196/43702)
Supplement: Multimedia Appendix 4 [file resprot_v12i1e43702_app4.pdf]

1R01AG076364-01 Betz, Marian

RESUME AND SUMMARY OF DISCUSSION: This application proposes a study to test the efficacy of Safety in Dementia (SID), an online decision aid that helps caregivers clarify values and preferences and implement their preferred options, on firearm safety decision quality and behaviors among a national sample of informal caregivers. This project has the potential impact of promoting firearm injury prevention among older adults with Alzheimer’s disease and related dementias (ADRD) through a person-centered approach that is respectful and supportive of their independence, autonomy, and rights, as well as supporting caregivers in addressing sensitive home safety issues. The investigative team is excellent with multidisciplinary and complementary expertise in aging, injury prevention, and gun research and a strong track record of publications in those areas of research. The environment was considered strong, and the person-centered element of the intervention was considered highly innovative. During the discussion the panel noted several strengths of the approach, including the thoughtful use of qualitative methods to inform refinement and acceptability of the intervention as well as the use and comparison of different recruitment techniques. The reviewers discussed a few limitations related to the approach including the lack of specific details around attrition rates, model parameters, and effect sizes. Although there was some initial disagreement about these limitations, the panel reached consensus that these limitations were considered minor and did not significantly reduce the project’s potential impact. Overall, the panel agreed that this was an impressive application from an exceptional group of investigators and could have a high impact in the field of healthy aging and decision-making among individuals with ADRD.

DESCRIPTION (provided by applicant): Cognitive and behavioral changes that come with Alzheimer's Disease and related dementias (ADRD) can increase the risk of injury or death, including from firearms. Suicide is the primary injury risk from firearms for people with ADRD, but ADRD-related confusion or paranoia can put others at risk. In the United States, an estimated 33-60% of people with ADRD have a firearm in the home, and 38% of ADRD caregivers identify firearms as an issue to address. Yet firearm safety is rarely discussed with ADRD patients or caregivers, leaving some ADRD caregivers without guidance about when and how to address the sensitive topic. To address this gap, we developed “Safety in Dementia” (SiD) for informal caregivers to address firearm safety (R34MH113539-NIA suppl). Based on theory and international standards for development, SiD is an online decision aid that helps caregivers clarify values and preferences and then commit to implementing their preferred options. SiD had high acceptability in a small pilot trial, and we hypothesize SiD will increase informal caregivers' preparation and self-efficacy to make and implement decisions that effectively address firearm access, thereby reducing injury risk. Over a three-year period, our multi-disciplinary, established study team will conduct an online randomized trial of SiD with national recruitment and longitudinal follow-up, with recruitment of informal caregivers of community-dwelling adults with ADRD who have firearm access. Through this trial and qualitative interviews with stakeholders, we aim to: (1) test the efficacy of SiD on firearm safety decision quality and behaviors among a national sample of informal caregivers; (2) compare varied methods (social media/internet versus via relevant organizations) in reaching informal caregivers; and (3) explore stakeholder longitudinal experiences with SiD and firearm-related decisions, including recommendations for optimal SiD use. Our underlying hypotheses are that SiD will be significantly associated with higher immediate decision quality and subsequent action to reduce firearm access, and that caregiver populations will vary by method of contact (social media/internet versus relevant organizations). The proposed research will meet a critical need for effective, acceptable tools to help caregivers address firearm access in the context of ADRD, thereby helping reduce firearm injuries and deaths while respecting and promoting older adult independence, autonomy, and rights. The aging of the U.S. population underscores the urgency of these issues, and our proposed work will provide the scientific foundation for future

implementation and evaluation of person- centered tools in real-world settings, including through enhanced understanding of methods for reaching and engaging caregivers.

PUBLIC HEALTH RELEVANCE: Deciding when and how to reduce firearm access for a person with Alzheimer's Disease and related dementias (ADRD) is one of the most emotional and difficult issues informal caregivers face – yet it is one that many caregivers must address, given the prevalence of firearms in the homes of community dwelling people with ADRD. The online “Safety in Dementia” decision aid provides caregivers with respectful, practical assistance in working through the decision, thereby helping them take action to reduce firearm access and prevent unintentional or intentional injuries. Through this multi-disciplinary proposal, we will test “Safety in Dementia” in an online, nationwide trial with a diverse sample of caregivers, thereby improving our understanding of both the decision aid's effects and how to use various methods to reach and engage caregivers.

CRITIQUE 1

Significance: 1
Investigator(s): 1
Innovation: 2
Approach: 2
Environment: 1

Overall Impact: This study aims to reduce gun related suicides, injuries and other deaths among patients with dementia. The study goals are to determine the efficacy of a web-based caregiver decision aid intervention (SiD), with or without reminders, and to compare methods of reaching caregivers. The findings from Aims 1 & 2, combined with proposed work with stakeholders (Aim 3), could identify necessary refinements and result in high impact study. This application is outstanding in that the topic matter is increasingly important given the number of older adults with firearms who are at risk for Alzheimer's and other dementias. The authors demonstrate expertise and sensitivity about community dwelling older adults struggling with independence and gun ownership. The research team is outstanding. The pilot study informs this larger study. This study will disseminate, and test decision aid developed during pilot study. The online “Safety in Dementia” decision aid provides caregivers with respectful, practical assistance in working through the decision, thereby helping them take action to reduce firearm access and prevent unintentional or intentional injuries. The research team is multidisciplinary representing geriatricians, EM docs, injury control and firearm control experts, research methods experts. The investigators will test “Safety in Dementia” in an online, nationwide trial with a diverse sample of caregivers, thereby improving our understanding of both the decision aid’s effects and how to use various methods to reach and engage caregivers. The investigators have identified an important population at risk for gun deaths and injuries and they have developed a clever intervention that does not take guns away from a highly armed population but engages patients and caregivers in discussions to make a gun safety plan. Only concerns are national representation in disseminating intervention and research follow up with care providers, requires further explanation of block randomization stratified by recruitment groups.

1. Significance:

Strengths

- Gun ownership in and of itself is problematic as it results in increased likelihood of suicide and deaths to others. Then throw in the fact that overall, Americans are aging and living longer

increasing risk of dementias. Finally, these individuals are already dealing with the loss of autonomy but should not be accessing and owning guns. The intervention developed during a pilot study is innovative in the investigators are finding ways to screen and to make gun safety plans with a neglected population.

- Older adults more likely to own guns.
- Older adults at higher risk for gun deaths and injury due to dementias.
- Caregivers have significant stress and must make important decisions about the person they care for including decisions about what to do with the guns.
- Decision aids increasingly are being developed to engage patients and their caregivers in patient care.

Weaknesses

- None noted.

2. Investigator(s):

Strengths

- The lead investigator is trained in both aging and injury prevention as well as gun control. Moreover, she has worked with some of the leading gun control experts in America.
- The Co-investigator is also an EM doc and is well trained in injury control and gun control in specific.
- The Co-Is are well trained in aging and injury control.
- All investigators are research active with specific expertise for this proposed study.
- Research team has experience working together on research and publications.

Weaknesses

- None noted.

3. Innovation:

Strengths

- Apolitical, respectful, kind firearm injury intervention that would positively resonate with various populations.
- Gun respect is going to have more reach.
- Easy access to decision support tool on the internet.
- Multidisciplinary research team.

Weaknesses

- Those at higher risk may not have internet access or be uncomfortable with the internet.
- Unclear if qualitative methods will be available to representative end users.

4. Approach:

Strengths

- RCT to determine efficacy and reach.

- Metrics on both providers and patients.
- Investigators are aware of Latinx heterogeneity.
- The screening tool is evidence based.
- Key study measures are empirically tested.
- RCT design.
- Data collection and measurement plans meet CONSORT guidelines.
- Based on a theoretical framework.
- Power analysis is correct.
- Plan for missing data.
- Testing delivery in Aim 2 on caregivers.
- Involvement of caregivers in study.
- Qualitative methods will inform the acceptability of this intervention and also find areas for improvement. Mixed methods studies are superior for innovative interventions.
- Longitudinal design to discover the long-term outcomes of the intervention.

Weaknesses

- How will we know if the sample size of 500 will be nationally representative?
- The investigators describe national reach via various communication strategies. Again, will it be representative?

5. Environment:

Strengths

- Colorado and Brown have excellent research resources and are both known for injury prevention research.

Weaknesses

- None noted.

Study Timeline:

Strengths

- Appropriately documented.

Weaknesses

- Should include missing data solutions.
- Should include intervention implementation and timeframe to begin study.

Protections for Human Subjects:

Acceptable Risks and/or Adequate Protections

Data and Safety Monitoring Plan (Applicable for Clinical Trials Only):

Acceptable

Inclusion Plans:

- Sex/Gender: Distribution justified scientifically
- Race/Ethnicity: Distribution justified scientifically
- For NIH-Defined Phase III trials, Plans for valid design and analysis: Not applicable
- Inclusion/Exclusion Based on Age: Distribution justified scientifically

Vertebrate Animals:
Not Applicable (No Vertebrate Animals)

Biohazards:
Not Applicable (No Biohazards)

Applications from Foreign Organizations:
Justified

Select Agents:
Acceptable

Resource Sharing Plans:
Acceptable

Budget and Period of Support:
Recommend as Requested

CRITIQUE 2

Significance: 3
Investigator(s): 2
Innovation: 4
Approach: 6
Environment: 3

Overall Impact: Safety in Dementia (SiD) is an online decision aid that helps caregivers clarify values and preferences and then commit to implementing their preferred options. Over a three-year period, the multi-disciplinary, established study team will conduct an online randomized trial of SiD with national recruitment and longitudinal follow-up, with recruitment of informal caregivers of community-dwelling adults with ADRD who have firearm access. While the topic is timely and relevant, the most-case scenario for impact from this proposal appears limited in terms of clinical utility, and precise measures of success could have been spelled out more clearly. Moreover, prevention strategies to keep long-

term motivation and prevent drop-outs from the longitudinal arm would have strengthened the overall research program.

1. Significance:

Strengths

- Estimated 33-60% of people with ADRD have a firearm in the home.
- About 5.8 million Americans – roughly 10% of all adults aged 65 years or older – are living with Alzheimer's disease and related dementias.
- Approximately one third of older Americans (>65 years) own a gun.
- 91% of older adult firearm deaths are due to suicide.

Weaknesses

- Quantifiable success metrics could have been more precisely defined.

2. Investigator(s):

Strengths

- PI has MD background with specialization in Emergency Medicine.
- Has experience in leading NIH-R01 grants.
- Worked with numerous national organizations on issues of injury prevention and healthy aging, including the Suicide Prevention Resource Center, the American Foundation for Suicide Prevention, and the American Geriatrics Society.
- Elected Member-at-Large (2012-14), President-Elect (2013-14) and President (2014-15), Academy of Geriatrics in Emergency Medicine, Society for Academic Emergency Medicine.
- Multi-year track record in publishing on aging and Alzheimer's disease in internally recognized peer-reviewed journals.

Weaknesses

- None noted.

3. Innovation:

Strengths

- Randomized clinical trials are among the sharpest tools in the arsenal of biomedical investigators.

Weaknesses

- Innovation section read mostly like another Significance section; it remains less clear to this reviewer how the present proposal goes beyond previous related approaches, or how it deviates precisely from the status quo.
- It remains underspecified what the proposed tools and intervention strategies exactly are.

4. Approach:

Strengths

- Already completed a pilot trial of SiD among adult caregivers of community-dwelling people with ADRD (n=209).
- Determine the efficacy of a web-based caregiver decision aid intervention (SiD), with or without reminders, and to compare methods of reaching caregivers.
- Bilingual approach: English + Spanish.

Weaknesses

- Online randomized controlled trial is geographically constrained, which makes it unclear how well the findings and conclusions can be informative about other parts of the US, or other countries.
- Estimated length of the questionnaires does not appear to be mentioned.
- More consideration could have been devoted to the expected drop out rates, and how to prevent them.
- ADRDs are known to feature a variety of sex-specific features, which could have been more comprehensively anticipated and directly analyzed at all stages of the approach.
- While linear mixed models are mentioned as a target statistical approach, it remains underdefined what random and what fixed effects the investigators plan to analyze to warrant this choice of model class in better answering the research goals, or which model specification will contain which covariates of no interest.
- Preliminary data: it is unclear what the nature of the computed effect size is: Cohen's d?

5. Environment:

Strengths

- Interdisciplinary team with established investigators with complementary skill sets.
- Dr. Betz has built a program of federally-funded research in firearm-related research at UCD (with Co-Is Knoepke, Matlock, and Nearing), where she leads the Firearm Injury Prevention Initiative.
- Expert Advisory Panel includes a firearms instructor and the Senior Communications Director for the National Shooting Sports Foundation (trade association for the firearm industry.
- Dr. Betz currently serves as PI on a multi-site randomized trial with longitudinal follow-up of older drivers and family members.

Weaknesses

- None noted.

Study Timeline:

Strengths

- Timeline is appropriate.

Weaknesses

- None noted.

Protections for Human Subjects:

Acceptable Risks and/or Adequate Protections

Data and Safety Monitoring Plan (Applicable for Clinical Trials Only):

Acceptable

Inclusion Plans:

- Sex/Gender: Distribution justified scientifically
- Race/Ethnicity: Distribution justified scientifically
- For NIH-Defined Phase III trials, Plans for valid design and analysis: Not applicable
- Inclusion/Exclusion Based on Age: Distribution justified scientifically

Vertebrate Animals:

Not Applicable (No Vertebrate Animals)

Biohazards:

Not Applicable (No Biohazards)

Resource Sharing Plans:

Acceptable

Budget and Period of Support:

Recommend as Requested

CRITIQUE 3

Significance: 1
Investigator(s): 1
Innovation: 1
Approach: 1
Environment: 1

Overall Impact: This project proposes to study the efficacy of a previously developed tool for helping caregivers of individuals with dementia decide how to deal with potentially injury causing items including firearms. The applicants prior work found that 40% of caregivers have safety concerns regarding firearms and are looking for assistance. Further, the tool they developed allows individuals to consider their own values and does not impose a single solution (e.g., remove all firearms from the home). The evaluation will consider both immediate and long-term outcomes. The investigation will study the efficacy of the tool as well as compare recruiting methods (social media ads versus interest groups). The team is experienced with complementary skills that cover all aspects of the proposed project. This is a strong well written application. I identified no weaknesses.

1. Significance:

Strengths

- Based on the applicant's preliminary work 40% of ADRD caregivers have safety concerns regarding firearms and 30% are look for options regarding firearm access. Therefore, testing the efficacy of a tool that they previously developed to address this issue could have a significant impact.

Weaknesses

- None noted.

2. Investigator(s):

Strengths

- The team has the relevant experience to complete this project and they have complementary skills and previous experience working together.
- The table illustrating the needed versus processed skills was very useful.

Weaknesses

- None noted.

3. Innovation:

Strengths

- The tool was developed with gun owners and is designed to be culturally sensitive and allow individuals to make choices based on their values. It does not advocate for a single solution.
- The work is designed with dissemination in mind by creating an accessible tool.

Weaknesses

- None noted.

4. Approach:

Strengths

- Testing the efficacy of a previously developed tool using RCT methodology.
- Will use and compare two recruitment techniques social media ads and interest groups. This will likely lead to interesting information that can be used to enhance this work and likely other firearm related work.
- The recruiting methods allow for low-cost national level recruitment. This will enhance generalizability and the potential impact of the work.

Weaknesses

- None noted.

5. Environment:

Strengths

- Strong environment with good resources to complete the work.

Weaknesses

- None noted.

Study Timeline:

Strengths

- The timeline accounts for dissemination and appears reasonable.

Weaknesses

- None noted.

Protections for Human Subjects:

Acceptable Risks and/or Adequate Protections

- The plan is appropriate.

Data and Safety Monitoring Plan (Applicable for Clinical Trials Only):

Acceptable

- Minimal risk.

Inclusion Plans:

- Sex/Gender: Distribution justified scientifically
- Race/Ethnicity: Distribution justified scientifically
- For NIH-Defined Phase III trials, Plans for valid design and analysis: Not applicable
- Inclusion/Exclusion Based on Age: Distribution justified scientifically
- Appear to be reasonable.

Vertebrate Animals:

Not Applicable (No Vertebrate Animals)

Biohazards:

Not Applicable (No Biohazards)

Resource Sharing Plans:

Not Applicable (No Relevant Resources)

Budget and Period of Support:

Recommend as Requested

THE FOLLOWING SECTIONS WERE PREPARED BY THE SCIENTIFIC REVIEW OFFICER TO SUMMARIZE THE OUTCOME OF DISCUSSIONS OF THE REVIEW COMMITTEE, OR REVIEWERS' WRITTEN CRITIQUES, ON THE FOLLOWING ISSUES:

PROTECTION OF HUMAN SUBJECTS: ACCEPTABLE

INCLUSION OF WOMEN PLAN: ACCEPTABLE

INCLUSION OF MINORITIES PLAN: ACCEPTABLE

INCLUSION ACROSS THE LIFESPAN: ACCEPTABLE

COMMITTEE BUDGET RECOMMENDATIONS: The budget was recommended as requested.

Footnotes for 1 R01 AG076364-01; PI Name: Betz, Marian Elizabeth

NIH has modified its policy regarding the receipt of resubmissions (amended applications).See Guide Notice NOT-OD-18-197 at https://grants.nih.gov/grants/guide/notice-files/NOT-OD-18-197.html. The impact/priority score is calculated after discussion of an application by averaging the overall scores (1-9) given by all voting reviewers on the committee and multiplying by 10. The criterion scores are submitted prior to the meeting by the individual reviewers assigned to an application, and are not discussed specifically at the review meeting or calculated into the overall impact score. Some applications also receive a percentile ranking. For details on the review process, see http://grants.nih.gov/grants/peer_review_process.htm#scoring.
